# Striatal Cholinergic Interneurons in a Knock-in Mouse Model of L-DOPA-Responsive Dystonia

**DOI:** 10.3389/fnsys.2018.00028

**Published:** 2018-06-27

**Authors:** Gul Yalcin-Cakmakli, Samuel J. Rose, Rosa M. Villalba, Lagena Williams, Hyder A. Jinnah, Ellen J. Hess, Yoland Smith

**Affiliations:** ^1^Yerkes National Primate Research Center, Emory University, Atlanta, GA, United States; ^2^Department of Pharmacology, Emory University, Atlanta, GA, United States; ^3^Department of Neurology, Emory University, Atlanta, GA, United States

**Keywords:** acetylcholine, striatum, parvalbumin, muscarinic receptor, aging

## Abstract

Striatal cholinergic dysfunction is a common phenotype associated with various forms of dystonia in which anti-cholinergic drugs have some therapeutic benefits. However, the underlying substrate of striatal cholinergic defects in dystonia remain poorly understood. In this study, we used a recently developed knock-in mouse model of dopamine-responsive dystonia (DRD) with strong symptomatic responses to anti-cholinergic drugs, to assess changes in the prevalence and morphology of striatal cholinergic interneurons (ChIs) in a model of generalized dystonia. Unbiased stereological neuronal counts and Sholl analysis were used to address these issues. To determine the potential effect of aging on the number of ChIs, both young (3 months old) and aged (15 months old) mice were used. For purpose of comparisons with ChIs, the number of GABAergic parvalbumin (PV)-immunoreactive striatal interneurons was also quantified in young mice. Overall, no significant change in the prevalence of ChIs and PV-immunoreactive cells was found throughout various functional regions of the striatum in young DRD mice. Similar results were found for ChIs in aged animals. Subtle changes in the extent and complexity of the dendritic tree of ChIs were found in middle and caudal regions of the striatum in DRD mice. Additional immunohistochemical data also suggested lack of significant change in the expression of striatal cholinergic M1 and M4 muscarinic receptors immunoreactivity in DRD mice. Thus, together with our previous data from a knock-in mouse model of DYT-1 dystonia (Song et al., 2013), our data further suggest that the dysregulation of striatal cholinergic transmission in dystonia is not associated with major neuroplastic changes in the morphology or prevalence of striatal ChIs.

**Highlights**

- There is no significant change in the number of striatal ChIs in young and aged mice model of DRD
- There is no significant change in the prevalence of striatal GABAergic PV-containing interneurons in the striatum of young mice models of DRD
- Subtle morphological changes in the dendritic arborization of striatal ChIs are found in the middle and caudal tiers of the striatum in young mice models of DRD
- The levels of both M1 and M4 muscarinic receptors immunoreactivity are not significantly changed in the striatum of DRD mice
- Major changes in the prevalence and morphology of striatal ChIs are unlikely to underlie striatal cholinergic dysfunction in DRD

## Introduction

Dystonia is the third most common movement disorder after essential tremor and Parkinson’s disease (Defazio, 2010). It is characterized by sustained or intermittent, involuntary twisting muscle contractions that lead to abnormal movements, postures or both (Albanese et al., 2013). Although most forms of dystonia are not associated with major structural changes and neuronal death, various hypotheses about the pathophysiology of dystonia have been proposed (Vitek, 2002; Breakefield et al., 2008; Calabresi et al., 2016; Jinnah et al., 2017; Jinnah and Hess, 2018). It is considered as a network disorder that affects basal ganglia and cerebellar outflow to the motor cortex. Dysregulation of striatal dopaminergic and cholinergic systems are commonly seen as key underlying substrates of various forms of dystonia (Pisani et al., 2007; Breakefield et al., 2008; Zhang et al., 2012; Eskow Jaunarajs et al., 2015; Pappas et al., 2015; Scarduzio et al., 2017).

Until recently, animal models of dystonia were either etiologic or symptomatic. Etiologic models usually recapitulate genetic forms of dystonia, like DYT-1 dystonia, but in many cases these animals do not display dystonia. On the other hand, many symptomatic animal models exhibit dystonic motor features that are induced by drug treatments or other interventions which are not directly associated with the underlying causes of dystonia in humans (Neychev et al., 2011; Wilson and Hess, 2013). However, Hess and colleagues have recently developed a knock-in mouse model that carries a causal mutation of dopamine-responsive dystonia (DRD) in humans. As young adults, these mice display the core features of human DRD, including dystonia that worsens throughout the day and improves in response to both L-DOPA and anti-cholinergic trihexyphenidyl treatment (Rose et al., 2015). However, as these mice age, dystonic movements wane, locomotor activity decreases and movement initiation becomes slower, without any evidence of midbrain dopaminergic neurons degeneration (Rose et al., 2017). So this model, which exhibits face, construct and predictive validity, provides an excellent tool to study possible structural, neurochemical and pathophysiological changes associated with DRD in young and old age.

In light of strong evidence that striatal cholinergic interneurons (ChIs) play a central role in the pathophysiology of dystonia (Pisani et al., 2007; Breakefield et al., 2008; Eskow Jaunarajs et al., 2015; Pappas et al., 2015), combined with the fact that anti-cholinergic drugs are the first-choice and the most effective symptomatic treatment for most forms of dystonia (Jankovic, 2006, 2013; Horn and Comella, 2007), an in-depth understanding of the anatomical and functional integrity of the intrastriatal cholinergic system in models of dystonia is needed.

Through the use of DYT-1 mice models, evidence for a hyper-cholinergic state that hampers long term synaptic plasticity through over-activation of M1 cholinergic receptors on striatal projection neurons, has been demonstrated (Martella et al., 2009; Sciamanna et al., 2012a,b; Maltese et al., 2014). Paradoxically, striatal cholinergic cell loss has been reported in a different mouse model of DYT1 dystonia (Pappas et al., 2015), but not in others (Song et al., 2013). To further assess potential changes in the striatal cholinergic system in other models of dystonia, we quantified the total number and studied the morphology of striatal ChIs in young and old DRD mice. We also used immunohistochemistry to determine if the expression of M1 and M4 muscarinic cholinergic receptors was altered in the striatum of DRD mice.

## Materials and Methods

### Animals

A total of 18 (10 males, eight females) adult (3 and 15 months old) mice were used in this study. This study was carried out in accordance with the recommendations of the National Institutes of Health Guidelines for Use of Animals in Research. The housing conditions and all experimental procedures performed in these animals were approved by the Emory University’s Institutional Animal Care and Use Committee. These mice belong to two groups: mice homozygous for the c.1160C>A TH mutation, which is homologous to human DRD causing TH mutation p.381Q>K, and normal littermates on a mixed C57BL/6J and DBA/2J background were bred, genotyped and maintained as previously described (Rose et al., 2015).

### Immunohistochemistry

At the time of euthanasia, animals were deeply anesthetized with an overdose of isoflurane (2% inhalation). The brain tissue was prepared by perfusion fixation with a 4% paraformaldehyde solution. After perfusion, the brain was post-fixed for 24 h at 4°C in the fixative solution before being cut in transverse sections (30 μm-thick) with a freezing microtome. Serial striatal brain sections from 3-month old and 15-month old wild-type (WT; *n* = 9, four females and five males) and DRD (*n* = 9, four females and five males) mice were immunostained with choline acetyltransferase (ChAT) antibody (ProSci, USA) at 1:200 dilution. Every 6th section, extending approximately from IA (interaural) 5.1 to IA 2.5 mm (Paxinos and Franklin, 2001), was used for stereological counts of ChAT-positive neurons. Striatal brain sections from 3-month old WT (*n* = 3) or DRD (*n* = 3) animals were immunostained with parvalbumin (PV) antibody (PV 27, SWANT, Switzerland) used at 1:15,000 dilution.

All primary antibodies have been extensively characterized and carefully tested for specificity in previous studies (Table 1). For immunohistochemistry procedures, sections were treated with 1% sodium borohydride for 20 min followed by a pre-incubation for 1 h in a solution containing 1% normal non-immune horse (for ChAT immunoreactions) or goat (for PV immunoreactions) serum, 0.3% Triton- X-100, and 1% bovine serum albumin (BSA) in PBS. Sections were then incubated for 24 h at room temperature (RT) in a solution containing the subsequent primary antibodies in 1% normal serum, 0.3% Triton-X-100 and 1% BSA in PBS. On the second day, sections were thoroughly rinsed in PBS and then incubated in a PBS solution containing either (secondary) biotinylated horse anti-goat IgGs (for ChAT staining) or goat anti-rabbit IgGs (for PV staining; 1:200; Vector, Burlingame, CA, USA) combined with 1% normal serum, 0.3% Triton-X-100 and 1% BSA for 90 min at RT. Sections were exposed to an avidin-biotin-peroxidase complex (ABC; 1:100; Vector) for 90 min followed by rinses in PBS and Tris buffer (50 mM; pH 7.6). Sections were then incubated within a solution containing 0.025% 3,3′-diamino-benzidine tetrahydrochloride (DAB; Sigma, St. Louis, MO, USA), 10 mM imidazole, and 0.005% hydrogen peroxide in TRIS buffer for 10 min at RT, rinsed with PBS, placed onto gelatin-coated slides, and coverslipped with Permount.

**Table 1:** Sources of primary antibodies used in the study.

Antibody	Immunogen	Manufacturer data	Dilution
Choline acetyltransferase	Purified human placental enzyme	Pro Sci (50–265), goat polyclonal	1:100
Parvalbumin	Purified Carp-II parvalbumin from muscle; directed against an epitope at the 1st calcium binding site	Swant (PV27), rabbit polyclonal	1:15,000
Muscarinic receptor 1	Muscarinic i3 loop fusion protein	Levey et al. (1991), rabbit polyclonal	1:100
Muscarinic receptor 4	Muscarinic i3 loop fusion protein	Levey et al. (1991), rabbit polyclonal	1:500

To label muscarinic receptors, M1 and M4 antibodies were kindly provided by Dr A. Levey (Emory University, GA, USA). The specificity tests, working dilution and incubation conditions for these antibodies were described previously (Levey et al., 1991; see Table 1). Antigen retrieval was applied to the tissue for M1 and M4 localization. For this procedure, sections were first rinsed thoroughly in 0.1 M PB buffer (pH 7.4) followed by an incubation for 30 min in 10 mM sodium citrate buffer (pH 8.5) preheated to 80°C in water bath and maintained at this temperature during the whole incubation period. The sections were kept in this solution until it cooled down to RT, then they were rinsed in 0.1 M PB (pH 7.4) and immersed in 2% non-fat dry milk in 0.1 M PB (pH 7.4) containing 0.3% Triton-X-100 and 0.01% sodium azide for 60 min (Jiao et al., 1999). This procedure was followed by the regular immunostaining protocol using the ABC immunoperoxidase method and DAB as chromogen. The incubation conditions were the same as those described above for the ChAT and PV antibody reactions.

### Stereological Cell Counts

A CCD camera (Leica DC 500; Leica IM50 software) attached to a Leica DMRB light microscope (Leica Microsystems, Inc., Bannockburn, IL, USA) connected to a computer with StereoInvestigator software (MicroBrightField, Williston, VT, USA) was used for stereological cell counts (West et al., 1991; West, 1999). Every 6th or 12th section extending from IA (interaural) 5.1 to IA 2.0 mm was used to count striatal ChAT-positive or PV-positive neurons, respectively.

**Figure 1:**
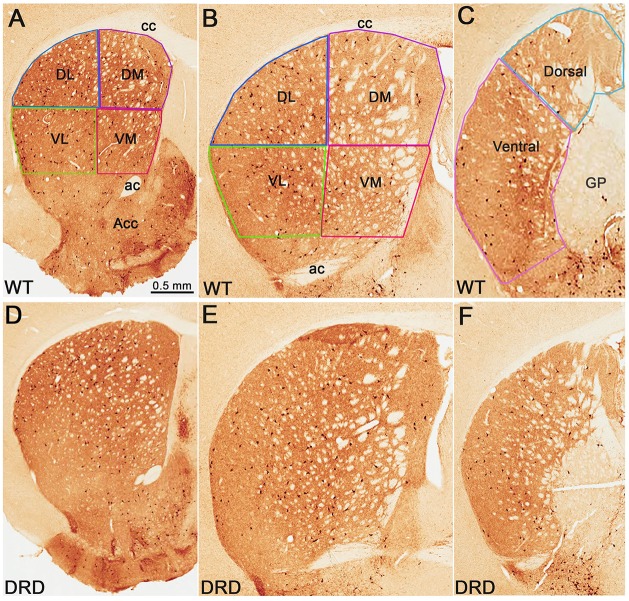
**(A–F)** Low power views of choline acetyltransferase (ChAT)-immunostained sections from wild-type (WT). **(A–C)** and dopamine-responsive dystonia (DRD) **(D–F)** mice at the rostral **(A,D)**, middle **(B,E)** and post-commissural **(C,F)** striatal levels. In the top row, the regions of interest in the dorsal striatum (DS) from which data were collected, are delineated. Abbreviations: ac, anterior commissure; acc, nucleus accumbens; cc, corpus callosum; DL, dorsolateral; DM, dorsomedial; GP, globus pallidus; VL, ventrolateral; VM, ventromedial. Scale bar in **(A)** valid for **(B–F)**.

For the evaluation of changes in the number of ChAT-positive neurons in specific functional territories of the dorsal striatum (DS), the structure was divided into rostral, middle and caudal/post-commissural tiers according to the mouse brain atlas (Paxinos and Franklin, 2001). From the rostral and middle striatal tiers, dorsolateral (DL), dorsomedial (DM), ventrolateral (VL), ventromedial (VM) quadrants were delineated, while the post-commissural tier was divided into a dorsal and a ventral sectors (Figure 1). The whole striatum, as well as striatal quadrants and divisions, were delineated at 2.5×. Counts of neuronal profiles were performed at 100× oil-immersion objective, at a 14 μm depth and 2 μm top guard zone by using optical fractionator probe of StereoInvestigator software. The counting frame was 150 × 150 μm in a sampling grid of 250 × 250 μm for ChAT-positive neurons count; 175 × 175 μm counting frame in 450 × 450 μm sampling grid for PV-positive neurons count. Gundersen coefficient of error (CE) was ~0.1 (0.05–0.17) for all regions of interest, except for the dorsal post-commissural region, being between 0.22 and 0.35.

### Striatal Volume Measurements

The Cavalieri’s principle was used to estimate striatal volumes (Gundersen and Osterby, 1981; Schmitz and Hof, 2005). The volume was determined by multiplying the sum of the ROI areas (striatum) by the distance between sections. The mean section thickness was estimated from at least four measurements/section, obtained by moving the focus from the top to the bottom surface of the tissue at each microscopic field with the *z*-axis position encoder of the stereology system.

### ChAT-Positive Cells’ Volume Measurements

The volume of ChAT-positive cell bodies was determined using the optical rotator probe of StereoInvestigator with a 3 μm focal plane separation and a 4-grid line separation of 4 μm. The optical fractionator probe was used to select the cells for volume measurement as a systematic random sampling scheme. Six WT and DRD mice were used. The volume of 20 ChIs randomly selected in different regions of interest was measured in each animal.

### Sholl Analysis of Striatal ChAT-Positive Neurons Dendritic Tree

The Sholl analysis was used to determine the extent and complexity of the arborization of the dendritic tree of ChAT-positive neurons (Sholl, 1953). To avoid any bias in the selection of neurons to be examined, ChAT-positive cells were selected from the different rostro-caudal and quadrants/divisions of the striatum (two neurons from each specific region) using the optical fractionator probe of StereoInvestigator software. Using this approach, the dendritic arborization of 20 neurons from each animal (i.e., 120 neurons per genotype) was traced under a 40× objective using the NeuroLucida software (MicroBrightField, Williston, VT, USA) and analyzed with the NeuroExplorer software. Using a modified Sholl method, concentric circles, the radii of which increase by 10 μm, were centered on the soma of the ChAT-immunoreactive neurons (Sholl, 1953). The dendritic length and number of intersections formed by the dendritic branches were then determined according to the radius level.

### Striatal M1 and M4 Immunostaining Intensity Measurements

To assess the relative intensity of striatal M1 and M4 immunostaining, M1- and M4-immunostained sections were scanned at 20× using a ScanScope CS scanning system (Aperio Technologies, Vista, CA, USA), and digital images of the stained tissue slides containing different levels of striatum (rostral, middle and caudal) were obtained by ImageScope viewer software (Aperio) and imported into ImageJ (National Institutes of Health, Schneider et al., 2012) for optical density measurement. The images were converted into 16-bit grayscale format and inverted. To control for differences in background staining, the optical density measurement in the corpus callosum (cc) was subtracted from that obtained in striatal measurements. Measurements of the intensity of labeling were obtained in three sections at rostral, middle and caudal striatal levels from DRD (*n* = 3) and WT (*n* = 3) animals in the 3-month old group. In each striatal section, the intensity of M1 and M4 immunostaining was measured in all quadrants delineated in rostral, middle and post-commissural striatum (see Figure 1). The resulting intensity values were averaged in DRD and WT group for comparison.

### Statistical Analyses

The data collection was done blind to the animal genotypes. One-way analysis of variance (ANOVA) was used for the comparisons of ChAT- and PV-positive neuron numbers between groups. Mann-Whitney *U* test was applied to compare the dendritic length and number of branches in each concentric circle of Sholl. For all analyses, statistical significance was defined as *p* < 0.05.

## Results

### Number of Striatal ChIs in DRD Mice

**Figure 2:**
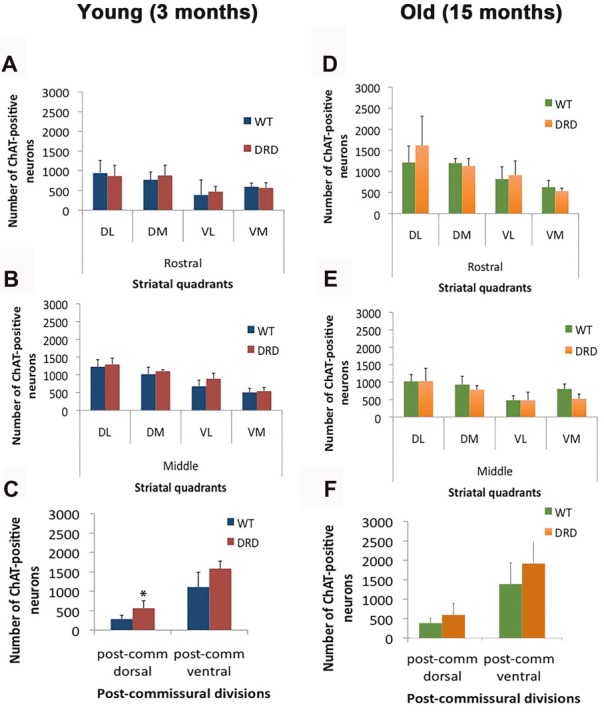
**(A–F)** Number of ChAT-positive neurons (average values ± SEM) in various quadrants and divisions of the rostral, middle and post-commissural tiers of the DS of young (3 months old; **A–C**) and old (15 months old; **D–F**) WT and DRD mice (*p < 0.05).

To determine if the total number of striatal ChIs was affected in DRD mice, unbiased stereological counts of ChAT-positive interneurons were collected from the DS in 3-month old DRD and WT mice. To assess possible regional heterogeneity in the number of ChAT-positive interneurons between the two groups, cell counts were collected from specific regions of interest in the rostral and caudal striatum (Figure 1). No significant difference was found in the total number of ChAT-positive neurons in any of the striatal regions examined, except in the dorsal division of the post-commissural striatum where a significantly larger number of ChAT-positive neurons was counted in DRD mice compared to controls (*p* < 0.05; Figures 1, 2A–C).

To assess possible effects of aging on cholinergic cell numbers in DRD, similar measurements were also collected from the striatum of 15-month old mice. In this group, no significant difference in ChAT-positive cell numbers was found in any striatal regions between DRD and WT animals (Figures 2D–F).

Using the Cavalieri’s principle, we also showed that there was no significant difference in striatal volume between the groups. In line with this observation, no significant difference in ChAT-positive neurons density was found between WT and DRD animals of both age groups (3 months old: WT 2036.06 ± 208.6/mm^3^ vs. DRD 2295.8 ± 264.4/mm^3^; 15 months old: WT 2004.4 ± 509.8/mm^3^ vs. DRD 2078.3 ± 382/mm^3^).

Overall, these findings demonstrate that there is no significant change in the number and density of ChAT-positive interneurons in the striatum of young and aged DRD mice.

### Number of Striatal Parvalbumin-Positive Interneurons in DRD Mice

**Figure 3:**
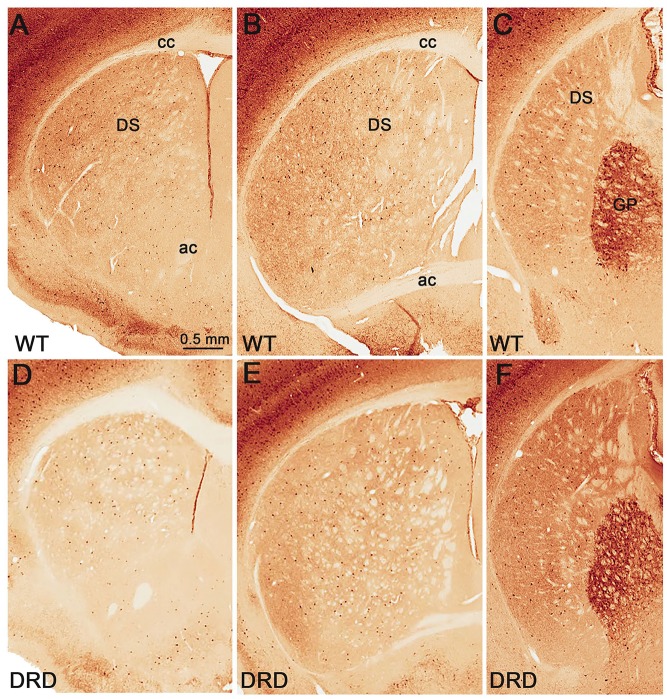
**(A–F)** Low power views of parvalbumin (PV)-immunostained sections from WT **(A–C)** and DRD **(D–F)** mice at the rostral **(A,D)**, middle **(B,E)** and post-commissural **(C,F)** striatal levels. Abbreviations: ac, anterior commissure; cc, corpus callosum; DS, dorsal striatum; GP, globus pallidus. Scale bar in **(A)** valid for **(B–F)**.

**Figure 4:**
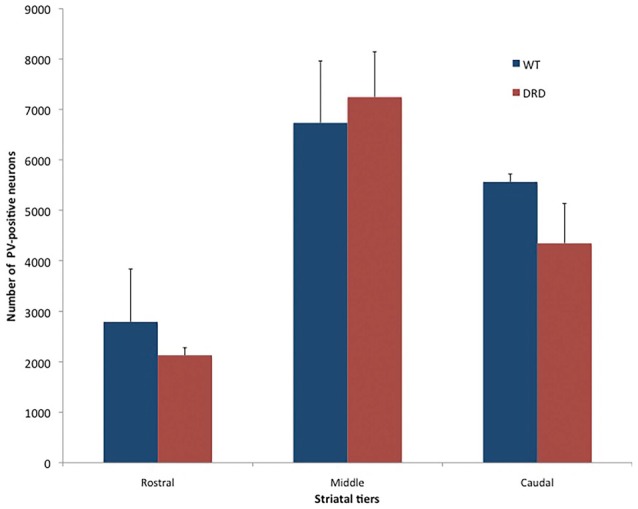
Number of PV-positive neurons (average values ± SEM) in the rostral, middle and caudal tiers of the DS of young WT and DRD mice. No significant difference was found between the two groups.

Because striatal PV-positive neuronal loss has been observed in other animal models of dystonia (Gernert et al., 2000; Reiner et al., 2013), we compared the total number of striatal PV-positive GABAergic interneurons in the DS of 3-month old DRD and WT mice (Figure 3). This study did not reveal any significant difference in the number of PV-positive neurons in rostral, middle and caudal tiers of the DS (Figure 4) between WT (*N* = 3; Total striatum: 12395 ± 1110.8) and DRD (*N* = 3; Total striatum: 13812.4 ± 1162.9) mice. Therefore, similar to ChAT-positive cells, there is no change in the number of PV-immunoreactive interneurons in the DS of DRD mice.

### Morphological Changes of Striatal ChIs in DRD Mice

**Figure 5:**
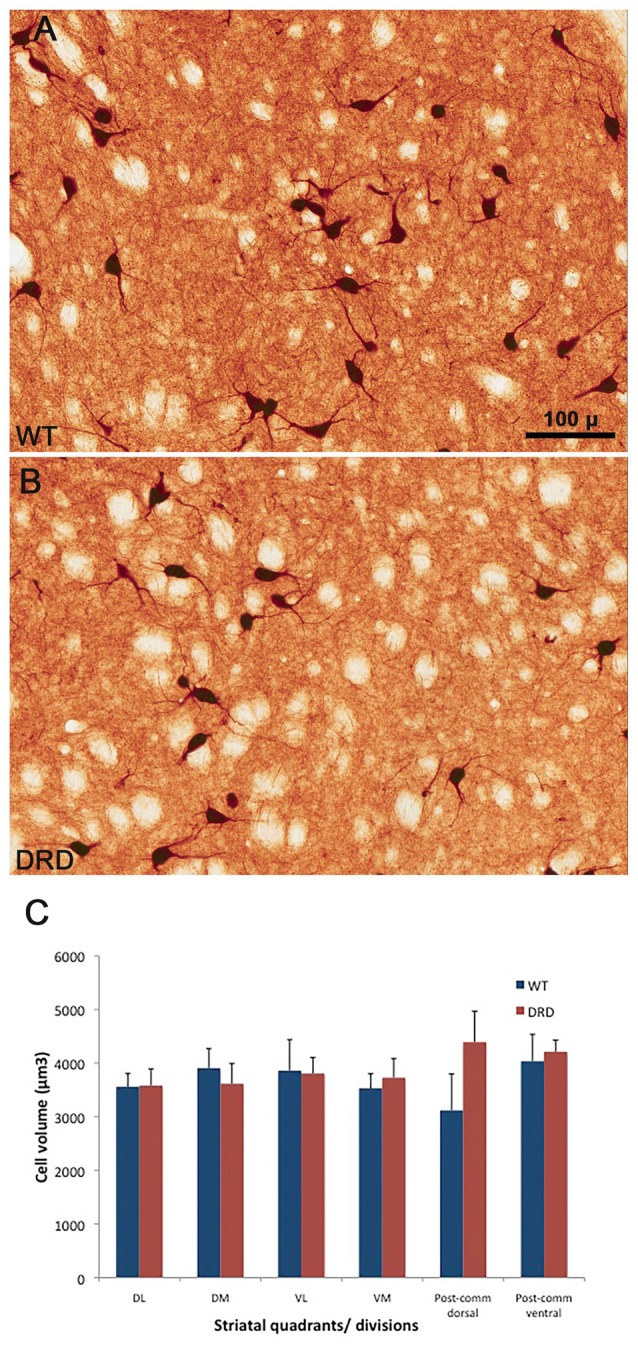
**(A–C)** High power views of ChAT-positive neurons in the DS of a WT **(A)** and a DRD **(B)** mouse to depict the morphological features of cholinergic interneurons (ChIs) between the two animal groups. **(C)** Compares the average (±SEM) volume of ChAT-immunoreactive perikarya measured from the different striatal regions of interest depicted in Figure 1. Three WT and three DRD mice were used in this analysis. The volume of 20 randomly selected striatal ChIs was measured in each mouse. No significant difference in ChIs perikaryal volume was found between the two animal groups in any of the striatal regions examined.

Based on recent findings showing that the somata of striatal ChAT-positive interneurons are slightly larger in a DYT-1 knock-in mouse model of dystonia than in WT mice (Song et al., 2013), we compared the perikarya volume of ChIs between DRD and WT mice. In contrast to the DYT-1 mice, our findings did not reveal any significant difference in striatal ChAT-positive cell body volume between DRD (3807.6 ± 147.7 μm^3^) and WT (3688.3 ± 174.3 μm^3^) mice (Figure 5). To ensure that this lack of morphological changes was a common feature of ChAT-positive neurons across all functional regions of the striatum, we compared the volume of ChAT-positive cell bodies randomly selected from the same quadrants and divisions used for the stereological counts. Similar to findings from the whole striatum, these observations did not reveal any significant difference in somata volumes between the two genotypes (Figure 5C).

**Figure 6:**
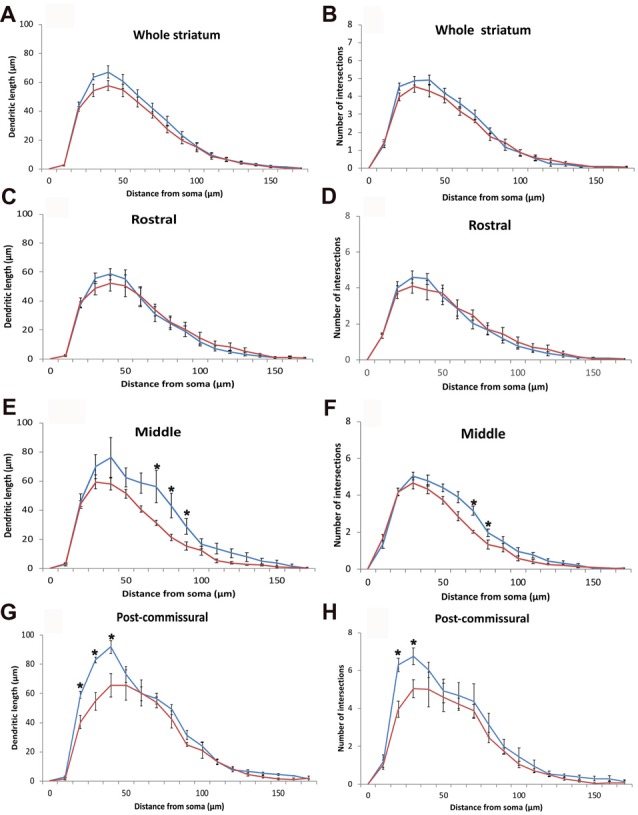
**(A–H)** Quantitative morphometric data (Mean ± SEM) from Sholl analysis about the length **(A,C,E,G)** and extent of arborization **(B,D,F,H)** of the dendritic tree of striatal ChIS according to incremental radii of 10 μm from the center of their perikaryon in WT (blue traces, *N* = 6, 20 cells/animal) and DRD (red traces, *N* = 6, 20 cells/animal) mice. Mean dendritic length and number of intersections made by the dendritic tree of ChAT-positive neurons were not significantly different in DRD mice compared to WT throughout all the concentric circles for the whole striatum **(A,B)**, but reached statistical significance in specific circles away from the soma in the middle **(E,F)** and post-commissural **(G,H)** tiers of the striatum (*p < 0.05).

To further assess possible morphological changes of ChAT-positive neurons between DRD and WT mice, we used the Sholl analysis to determine if there was a change in the extent and complexity of the dendritic arbor of ChAT-positive neurons between the two animal groups. For this analysis, 2 ChAT-positive neurons were randomly selected by the optical fractionator probe in each of the pre-determined quadrants and divisions of the striatum of 6 WT and 6 DRD mice in the 3-month old group (total: 20 neurons/animal). Although the mean dendritic length of ChAT-positive neurons and the number of intersections made by the dendritic tree were reduced in DRD mice compared to WT throughout all the concentric circles, this difference did not reach statistical significance when data from the whole striatum were examined (*p* = 0.053 for dendritic length and *p* = 0.065 for number of intersections, Figures 6A,B).

To search for possible regional differences in the morphology of the dendritic arbor of ChIs, dendritic lengths and numbers of intersections were measured for ChIS in each rostro-caudal tier of the striatum. There was no significant change in the rostral striatal region for both parameters (Figures 6C,D). However, in the middle tier of the striatum, the maximum extent of the dendritic tree was significantly reduced in the DRD group (109.6 μm in WT vs. 98.5 μm in DRD, *p* = 0.039) as were the total number of dendritic intersections (33.06 μm in WT vs. 28.71 μm in DRD, *p* = 0.041) and the number of intersections in the 7th and 8th concentric circle (*p* = 0.008 and *p* = 0.047, Figure 6F). Similarly, a decrease in the dendritic length in the 7th, 8th and 9th circles was detected (*p* = 0.048, 0.018 and 0.040 respectively, Figure 6E). In the post-commissural tier, the dendritic length and number of dendritic intersections in the 2nd and 3rd circles were significantly reduced in the DRD group compared to WT (for length *p* = 0.005, 0.004 and 0.009 for 4th circle; for intersections *p* = 0.001, 0.023, Figures 6G,H).

Further analysis for the quadrants and divisions of the striatum revealed that the loss of complexity in the dendritic tree of ChIs was more pronounced in the dorso-medial quadrant. In this region, the maximum extent of the dendritic tree (108.8 μm in WT vs. 90 μm in DRD, *p* = 0.028), the total dendritic length (390.8 μm in WT vs. 287.8 μm in DRD, *p* = 0.032) and the number of dendritic intersections (30.4 μm in WT vs. 21.5 μm in DRD, *p* = 0.008) were all significantly lower in the DRD group than in WT animals.

Overall, this morphological analysis revealed a significant reduction in the complexity of the dendritic arbor of striatal ChIs in DRD mice, being more prominent in the middle and post-commissural tiers and in the DM quadrant of the striatum.

### Striatal Expression of Muscarinic Receptors M1 and M4 Immunoreactivity in DRD Mice

**Figure 7:**
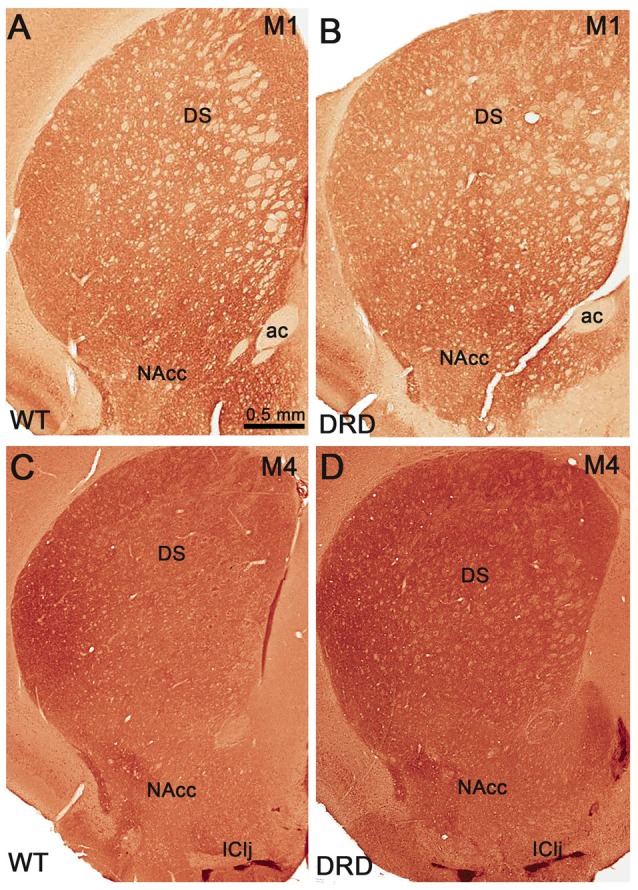
**(A–D)** Low power views of striatal sections that compare M1 **(A,B)** and M4 **(C,D)** muscarinic receptors immunoreactivity between WT **(A,C)** and DRD **(B,D)** mice. Abbreviations: ac, anterior commissure; DS, dorsal striatum; IClj, Island of Calleja; NAcc, nucleus accumbens. Scale bar in **(A)** valid for **(B–D)**.

**Figure 8:**
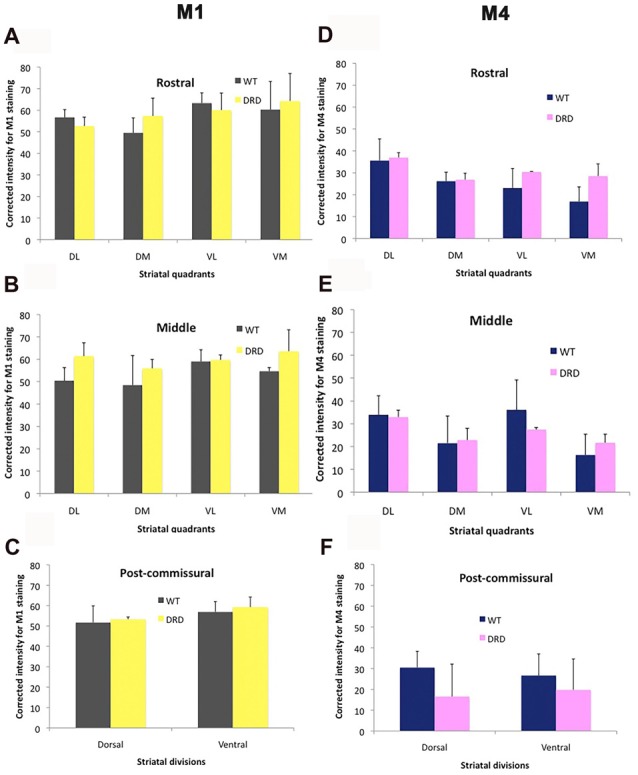
**(A–F)** Comparisons of M1 and M4 immunostaining intensity (Average ± SEM) in the different striatal regions depicted in Figure 1 between WT and DRD mice. No significant difference in staining intensity for either receptor subtype was found between the two animal groups.

To determine if changes in expression of cholinergic muscarinic receptors could potentially contribute to the striatal pathophysiology of DRD dystonia, we compared the intensity of M1 and M4 muscarinic receptors immunoreactivity in the striatum of 3-month old WT and DRD mice. Intensity measurements of M1/M4 immunostaining were collected from each quadrant and division of the striatum that were delineated for stereological count of ChAT-positive neurons. No significant change was detected in the pattern and intensity of labeling between WT (*n* = 3) and DRD mice (*n* = 3) in the young group (Figures 7, 8).

## Discussion

In light of strong evidence that striatal cholinergic dysfunction contributes to some aspects of dystonia pathophysiology, and the therapeutic benefit of anti-cholinergic drugs in patients with dystonia (Breakefield et al., 2008; Eskow Jaunarajs et al., 2015; Pappas et al., 2015), we looked at the prevalence and morphology of ChIs, and assessed possible changes in the expression level of M1 and M4 muscarinic receptors in the striatum of a newly developed knock-in mouse model of DRD that recapitulates the core clinical features and etiology of the human disease and responds to anti-cholinergic therapy (Rose et al., 2015). Our findings demonstrate that the number of ChIs is not altered in most striatal regions of DRD mice, except in the dorsal post-commissural tier where a slight increase in cholinergic cell number was found in 3-month old DRD animals. Similar findings were found in both 3-month old and 15-month old mice, indicating that aging does not have an impact on the number of striatal ChIs in DRD. Along the same line as ChIs, the total number of PV-positive GABAergic interneurons was not changed in the striatum of DRD mice. At the morphological level, striatal ChIs underwent subtle changes in the length and complexity of their dendritic tree but did not display any change in the size of their perikarya, in DRD mice. The M1 and M4 muscarinic receptor immunoreactivity was also not significantly altered in the striatum of DRD mice. Thus, together with our previous report that showed minor structural changes in various striatal cell types in a DYT-1 mouse model of dystonia (Song et al., 2013), our data suggest that the cholinergic imbalance seen in DRD, and other forms of dystonia, is unlikely to result from major morphological changes in striatal ChIs and gross expression level of M1/M4 muscarinic receptors. Further high-resolution studies that examine structural and functional changes at the synaptic level are needed to further understand the pathobiology and neurotransmitter dysregulation in various forms of dystonia.

### Prevalence and Morphology of Striatal Cholinergic Neurons in Young and Aged DRD Mice

As mentioned above, our findings did not reveal major change in the total number, and only subtle changes in the morphology, of ChIs in the striatum of DRD mice. To ensure that specific regional differences were not overlooked in this analysis, we counted striatal ChIs in various quadrants of the DS defined by their relationships with functionally distinct cortical areas. This analysis did not reveal major inter-regional differences in the counts of ChIs, except for a slight numerical increase in the dorsal part of the post-commissural striatum in 3-month old mice. The number of striatal ChIs has been assessed in other animal models of primary dystonia, specifically in different DYT-1 mouse models (Hamann et al., 2006; Sciamanna et al., 2012a; Song et al., 2013; Pappas et al., 2015). In line with our results, no change in the number of striatal ChIs was detected in most of these studies, except for the recent report of Pappas et al. (2015) which provides evidence for a 40% reduction in the number of ChIs in the DL striatum (i.e., sensorimotor territory) of a DYT-1 mouse model with a conditional deletion of torsinA gene in the embryonic progenitors of forebrain cholinergic and GABAergic neurons (Pappas et al., 2015). These mice show dystonic-like twisting movements responsive to anti-cholinergic treatment starting during the juvenile period (15–17 days postnatal), at a time which coincides with apoptotic death of striatal ChIs (Pappas et al., 2015). Paradoxically, despite more than 70% decrease in striatal extracellular acetylcholine in these mice, their motor deficits could be alleviated by muscarinic cholinergic antagonists, as is the case for some forms of primary human dystonia (Eskow Jaunarajs et al., 2015; Pappas et al., 2015). Because of the limited amount of findings from postmortem studies of brain pathology in dystonic patients (Albin et al., 2003; Standaert, 2011; Paudel et al., 2012), it remains unclear as to whether the loss or not of striatal ChIs better translates to the human condition. The paucity of reliable postmortem brain tissue from dystonia patients remains a major roadblock to advancement of knowledge in dystonia pathobiology and therapeutics.

Our findings revealed subtle, though significant, decrease in the extent and complexity of the dendritic tree of ChIs, without any change in their perikaryal size, in DRD mice. Those morphological changes are different from the recently reported increased somata size of ChIs in a knock-in model of DYT-1 dystonia, (Song et al., 2013). At present, the significance of this difference between the two dystonia mice models remains unclear. Although the larger perikaryal size of ChIs was suggested as a potential source of the increased striatal cholinergic tone in DYT-1 mice (Song et al., 2013; Pappas et al., 2015), this hypothesis remains to be tested. Similarly, the functional significance of the minor change noticed in the dendritic arbor of ChIs in DRD mice is currently unknown, although it may underlie some changes in the drive and modulation of these neurons by extrinsic GABAergic, glutamatergic and dopaminergic inputs, known to play a major role in regulating ChIs activity (Gonzales et al., 2013; Gonzales and Smith, 2015). Patch clamp recording and electron microscopic studies of ChIs in DRD animals are warranted to further address the anatomical and physiological consequences of these dendritic morphology changes on ChIs regulation.

In a recent study, Hess and colleagues demonstrated that the severity of dystonic movements is decreased, while the locomotor activity and movement initiation are slowed, in aged DRD mice (Rose et al., 2017). Despite this age-related development of hypokinetic behavior, these animals did not exhibit any significant degeneration of midbrain dopaminergic neurons, thereby providing evidence that DRD mice develop parkinsonian motor features with aging, an observation reminiscent of those reported from DRD patients, who display less dystonic symptoms, but more parkinsonian motor features with age (Tadic et al., 2012; Wijemanne and Jankovic, 2015; Rose et al., 2017). Because of this symptomatic difference between young and aged mice, we counted the number of ChAT-positive neurons in aged (15 months old) DRD animals and compared those with data from young (3 months old) DRD mice. This analysis did not reveal any significant loss of ChIs in the striatum of aged mice, suggesting that the dynamic evolution of the behavioral phenotype of the DRD mice from dystonia to parkinsonism does not rely on a mere change in the number of striatal ChIs.

### PV-Positive GABAergic Interneurons in the Striatum of DRD Mice

In addition to ChIs, the striatum contains a large variety of GABAergic interneurons characterized by their differential content in calcium binding proteins and electrophysiological properties (Kawaguchi, 1993; Kawaguchi et al., 1995; Tepper and Bolam, 2004; Tepper et al., 2010). Among those are the fast spiking PV-positive GABAergic cells, known to play a major role in feedforward inhibition of GABAergic spiny projection neurons (Tepper et al., 2008). Although our recent study did not reveal any significant difference in the number of PV-positive GABAergic interneurons in the striatum of DYT-1 mice (Song et al., 2013), data from the d^tsz^ hamster model of primary paroxysmal dystonia showed a significant and widespread loss, and maturation delay, of PV-positive striatal neurons (Gernert et al., 2000; Bode et al., 2017). Reiner et al. (2013) recently suggested that the progressive loss of PV-positive neurons in the motor striatum may determine the timing of dystonia onset in a mouse model of Huntington’ disease. Thus, the loss of striatal fast spiking interneurons may significantly contribute to the pathophysiology of some forms of dystonia. However, the fact that neither DRD nor DYT-1 (Song et al., 2013) mice exhibit any significant change in the number of PV-containing interneurons suggest that the developmental dysregulation of these neurons may differ between various dystonia phenotypes. Because of the limited amount of striatal tissue available from old mice, stereological counts of PV-positive interneurons could not be performed in this animal group. On the other hand, a lack of change in the number of PV-positive interneurons, does not necessarily rule out the possible involvement of these cells in DRD pathophysiology. Future electrophysiological studies looking at the baseline cellular properties and responses to extrinsic inputs of fast spiking PV-containing interneurons in control and DRD mice are needed to further address this issue.

### M1 and M4 Muscarinic Receptors in the Striatum of DRD Mice

Despite the potential importance of intrastriatal cholinergic dysfunction in dystonia, very little is known about expression levels of cholinergic receptors in the striatum in dystonia patients or animal models of dystonia (for a recent review see Eskow Jaunarajs et al., 2015). Thus, in an attempt to assess gross changes in cholinergic receptors’ expression in the striatum of DRD mice, we used immunohistochemistry to look at the striatal expression of M1 and M4 muscarinic receptors. Both M1 and M4 receptors are heavily expressed in the striatum where they mediate pre- and post-synaptic cholinergic functions (Pisani et al., 2007; Eskow Jaunarajs et al., 2015). Their importance in regulating intrastriatal cholinergic, dopaminergic and glutamatergic transmission and their impact upon long term synaptic plasticity of corticostriatal glutamatergic synapses have been demonstrated (see Pisani et al., 2007; for reviews, Eskow Jaunarajs et al., 2015). Because they are partly localized on different striatal cell types and afferents, coupled with the fact that they are linked to different G-proteins signaling cascades (M1: G_q/11_; M4: G_i/o_) that have opposite effects on neuronal excitability, potential alterations in their respective functional expression may underlie differential effects upon striatal projection neurons. Overall, the pattern of immunoreactivity for either receptor subtype was consistent with that reported in previous studies (Levey et al., 1991; Bernard et al., 1992, 1999; Hersch and Levey, 1995) using the same antibodies. Densitometry measurements of immunoreactivity did not reveal significant changes in M1 or M4 expression levels in the striatum of DRD mice. Such information could not be collected from old mice because of the restricted amount of striatal tissue available from these animals. Because of the limited amount of data available, it is difficult to determine if these findings are solely relevant to DRD or apply to other forms of dystonia. In that regard, a recent study showed that the expression of M1 receptors immunoreactivity is increased, while the [^3^H] pirenzepine binding (preferential M1 ligand) is decreased, in the DM striatum of the d^tsz^ hamster model of primary paroxysmal dystonia (Gernert et al., 2000; Hamann et al., 2017). Interestingly, this change was specific for M1 (i.e., no change in M4 expression) and confined to a striatal subregion where the activity of projection neurons was found to be increased in this animal model (Gernert et al., 2000; Hamann et al., 2017). Because anti-cholinergic agents do not have significant therapeutic benefits for dystonia in this model, it is difficult to relate these findings with those gathered from our DRD model, or any other models of dystonia, in which anti-cholinergic drugs alleviate dystonic symptoms (Dang et al., 2012; Jankovic, 2013; Maltese et al., 2014; Eskow Jaunarajs et al., 2015; Pappas et al., 2015; Rose et al., 2015). Despite this limitation, it is noteworthy that the morphological changes in the dendritic tree of ChIs were more pronounced in the DM striatum, in our study. Further studies are needed to reconcile these observations. It is important to note that the densitometry measurements of immunoreactivity used as an index of protein expression in both our study and that of Hamann et al. (2017) have some limitations in sensitivity that could partly contribute to these different results. Furthermore, results from both studies do not exclude possible alterations in pharmacological properties, subcellular localization and second messenger coupling of M1 and M4 receptors. Future studies are warranted to further address these issues.

## Concluding Remarks

Despite the general agreement that anti-cholinergic drugs are effective in alleviating dystonic symptoms in patients and animal models of various forms of generalized dystonia, the exact substrate of cholinergic dysfunction in dystonia remains poorly understood. In this study, we used a new model of DRD (Rose et al., 2015) which responds to anti-cholinergic therapy, to demonstrate that neither the prevalence nor the morphology of striatal ChIs are drastically affected in these animals. Similarly, we did not encounter major changes in M1 and M4 muscarinic receptors immunoreactivity in the striatum of these animals. Thus, together with our previous study in DYT-1 mice (Song et al., 2013), the present findings further highlight the fact that the neurochemical and neuronal network dysfunctions associated with dystonia are subtle and most likely confined to structural and functional changes of specific synaptic microcircuits. High resolution electron microscopic studies are in progress to assess the significance of such alterations in the striatum of our mouse model of DRD.

## Author Contributions

GY-C participated in study design, collected all data and prepared a draft of the written manuscript, SJR was involved in the development and characterization of the DRD mice and the write-up of part of the manuscript. RMV helped with the collection of stereological data presented in this article. LW helped with the collection of stereological data. HAJ and EJH was involved in the behavioral characterization of the DRD mice and the write-up of the manuscript. YS developed the study design, was involved in data analysis, participated in the write-up of the manuscript.

## Conflict of Interest Statement

The authors declare that the research was conducted in the absence of any commercial or financial relationships that could be construed as a potential conflict of interest.

## References

[B1] Albanese A. Bhatia K. Bressman S. B. Delong M. R. Fahn S. Fung V. S.. (2013). Phenomenology and classification of dystonia: a consensus update. Mov. Disord. 28, 863–873. 10.1002/mds.25475 23649720 PMC3729880

[B2] Albin R. L. Cross D. Cornblath W. T. Wald J. A. Wernette K. Frey K. A.. (2003). Diminished striatal [^123^I]iodobenzovesamicol binding in idiopathic cervical dystonia. Ann. Neurol. 53, 528–532. 10.1002/ana.10527 12666122

[B3] Bernard V. Levey A. I. Bloch B. (1999). Regulation of the subcellular distribution of m4 muscarinic acetylcholine receptors in striatal neurons *in vivo* by the cholinergic environment: evidence for regulation of cell surface receptors by endogenous and exogenous stimulation. J. Neurosci. 19, 10237–10249. 10.1523/jneurosci.19-23-10237.1999 10575021 PMC6782421

[B4] Bernard V. Normand E. Bloch B. (1992). Phenotypical characterization of the rat striatal neurons expressing muscarinic receptor genes. J. Neurosci. 12, 3591–3600. 10.1523/jneurosci.12-09-03591.1992 1527598 PMC6575743

[B5] Bode C. Richter F. Spröte C. Brigadski T. Bauer A. Fietz S.. (2017). Altered postnatal maturation of striatal GABAergic interneurons in a phenotypic animal model of dystonia. Exp. Neurol. 287, 44–53. 10.1016/j.expneurol.2016.10.013 27780732

[B6] Breakefield X. O. Blood A. J. Li Y. Hallett M. Hanson P. I. Standaert D. G. (2008). The pathophysiological basis of dystonias. Nat. Rev. Neurosci. 9, 222–234. 10.1038/nrn2337 18285800

[B7] Calabresi P. Pisani A. Rothwell J. Ghiglieri V. Obeso J. A. Picconi B. (2016). Hyperkinetic disorders and loss of synaptic downscaling. Nat. Neurosci. 19, 868–875. 10.1038/nn.4306 27351172

[B8] Dang M. T. Yokoi F. Cheetham C. C. Lu J. Vo V. Lovinger D. M.. (2012). An anticholinergic reverses motor control and corticostriatal LTD deficits in *Dyt1* ΔGAG knock-in mice. Behav. Brain Res. 226, 465–472. 10.1016/j.bbr.2011.10.002 21995941 PMC3225290

[B9] Defazio G. (2010). The epidemiology of primary dystonia: current evidence and perspectives. Eur. J. Neurol. 17, 9–14. 10.1111/j.1468-1331.2010.03053.x 20590802

[B10] Eskow Jaunarajs K. L. Bonsi P. Chesselet M. F. Standaert D. G. Pisani A. (2015). Striatal cholinergic dysfunction as a unifying theme in the pathophysiology of dystonia. Prog. Neurobiol. 127–128, 91–107. 10.1016/j.pneurobio.2015.02.002 25697043 PMC4420693

[B11] Gernert M. Hamann M. Bennay M. Loscher W. Richter A. (2000). Deficit of striatal parvalbumin-reactive GABAergic interneurons and decreased basal ganglia output in a genetic rodent model of idiopathic paroxysmal dystonia. J. Neurosci. 20, 7052–7058. 10.1523/jneurosci.20-18-07052.2000 10995851 PMC6772842

[B13] Gonzales K. K. Pare J. F. Wichmann T. Smith Y. (2013). GABAergic inputs from direct and indirect striatal projection neurons onto cholinergic interneurons in the primate putamen. J. Comp. Neurol. 521, 2502–2522. 10.1002/cne.23295 23296794 PMC3983787

[B12] Gonzales K. K. Smith Y. (2015). Cholinergic interneurons in the dorsal and ventral striatum: anatomical and functional considerations in normal and diseased conditions. Ann. N Y Acad. Sci. 1349, 1–45. 10.1111/nyas.12762 25876458 PMC4564338

[B14] Gundersen H. J. Osterby R. (1981). Optimizing sampling efficiency of stereological studies in biology: or ‘do more less well!’. J. Microsc. 121, 65–73. 10.1111/j.1365-2818.1981.tb01199.x 7014910

[B15] Hamann M. Plank J. Richter F. Bode C. Smiljanic S. Creed M.. (2017). Alterations of M1 and M4 acetylcholine receptors in the genetically dystonic (*dt*^sz^) hamster and moderate antidystonic efficacy of M1 and M4 anticholinergics. Neuroscience 357, 84–98. 10.1016/j.neuroscience.2017.05.051 28596119

[B16] Hamann M. Raymond R. Varughesi S. Nobrega J. N. Richter A. (2006). Acetylcholine receptor binding and cholinergic interneuron density are unaltered in a genetic animal model of primary paroxysmal dystonia. Brain Res. 1099, 176–182. 10.1016/j.brainres.2006.04.100 16764832

[B17] Hersch S. M. Levey A. I. (1995). Diverse pre- and post-synaptic expression of m1–m4 muscarinic receptor proteins in neurons and afferents in the rat neostriatum. Life Sci. 56, 931–938. 10.1016/0024-3205(95)00030-a 10188795

[B102] Horn S. Comella C. L. (2007). “Treatment of dystonia,” in Parkinson’s Disease & Movement Disorders, eds Jankovic J. Tolosa E. (Lippincott Williams: Wilkins), 348–355.

[B19] Jankovic J. (2006). Treatment of dystonia. Lancet Neurol. 5, 864–872. 10.1016/S1474-4422(06)70574-9 16987733

[B20] Jankovic J. (2013). Medical treatment of dystonia. Mov. Disord. 28, 1001–1012. 10.1002/mds.25552 23893456

[B21] Jiao Y. Sun Z. Lee T. Fusco F. R. Kimble T. D. Meade C. A.. (1999). A simple and sensitive antigen retrieval method for free-floating and slide-mounted tissue sections. J. Neurosci. Methods 93, 149–162. 10.1016/s0165-0270(99)00142-9 10634500

[B22] Jinnah H. A. Hess E. J. (2018). Evolving concepts in the pathogenesis of dystonia. Parkinsonism Relat. Disord. 46, S62–S65. 10.1016/j.parkreldis.2017.08.001 28784298 PMC5696051

[B23] Jinnah H. A. Neychev V. Hess E. J. (2017). The anatomical basis for dystonia: the motor network model. Tremor Other Hyperkinet. Mov. 7:506. 10.7916/D8V69X3S 29123945 PMC5673689

[B24] Kawaguchi Y. (1993). Physiological, morphological, and histochemical characterization of three classes of interneurons in rat neostriatum. J. Neurosci. 13, 4908–4923. 10.1523/jneurosci.13-11-04908.1993 7693897 PMC6576359

[B25] Kawaguchi Y. Wilson C. J. Augood S. J. Emson P. C. (1995). Striatal interneurones: chemical, physiological and morphological characterization. Trends Neurosci. 18, 527–535. 10.1016/0166-2236(95)98374-8 8638293

[B26] Levey A. I. Kitt C. A. Simonds W. F. Price D. L. Brann M. R. (1991). Identification and localization of muscarinic acetylcholine receptor proteins in brain with subtype-specific antibodies. J. Neurosci. 11, 3218–3226. 10.1523/jneurosci.11-10-03218.1991 1941081 PMC6575445

[B27] Maltese M. Martella G. Madeo G. Fagiolo I. Tassone A. Ponterio G.. (2014). Anticholinergic drugs rescue synaptic plasticity in DYT1 dystonia: role of M1 muscarinic receptors. Mov. Disord. 29, 1655–1665. 10.1002/mds.26009 25195914 PMC4216601

[B28] Martella G. Tassone A. Sciamanna G. Platania P. Cuomo D. Viscomi M. T.. (2009). Impairment of bidirectional synaptic plasticity in the striatum of a mouse model of DYT1 dystonia: role of endogenous acetylcholine. Brain 132, 2336–2349. 10.1093/brain/awp194 19641103 PMC2766181

[B29] Neychev V. K. Gross R. E. Lehéricy S. Hess E. J. Jinnah H. A. (2011). The functional neuroanatomy of dystonia. Neurobiol. Dis. 42, 185–201. 10.1016/j.nbd.2011.01.026 21303695 PMC3478782

[B30] Pappas S. S. Darr K. Holley S. M. Cepeda C. Mabrouk O. S. Wong J. M.. (2015). Forebrain deletion of the dystonia protein torsinA causes dystonic-like movements and loss of striatal cholinergic neurons. Elife 4:e08352. 10.7554/elife.08352 26052670 PMC4473728

[B31] Paudel R. Hardy J. Revesz T. Holton J. L. Houlden H. (2012). Review: genetics and neuropathology of primary pure dystonia. Neuropathol. Appl. Neurobiol. 38, 520–534. 10.1111/j.1365-2990.2012.01298.x 22897341

[B101] Paxinos G. Franklin K. J. (2001). The Mouse Brain in Stereotaxic Coordinates. California: Academic Press.

[B32] Pisani A. Bernardi G. Ding J. Surmeier D. J. (2007). Re-emergence of striatal cholinergic interneurons in movement disorders. Trends Neurosci. 30, 545–553. 10.1016/j.tins.2007.07.008 17904652

[B33] Reiner A. Shelby E. Wang H. Demarch Z. Deng Y. Guley N. H.. (2013). Striatal parvalbuminergic neurons are lost in Huntington’s disease: implications for dystonia. Mov. Disord. 28, 1691–1699. 10.1002/mds.25624 24014043 PMC3812318

[B34] Rose S. J. Harrast P. Donsante C. Fan X. Joers V. Tansey M. G.. (2017). Parkinsonism without dopamine neuron degeneration in aged L-dopa-responsive dystonia knockin mice. Mov. Disord. 32, 1694–1700. 10.1002/mds.27169 28949038 PMC5744486

[B35] Rose S. J. Yu X. Y. Heinzer A. K. Harrast P. Fan X. Raike R. S.. (2015). A new knock-in mouse model of L-DOPA-responsive dystonia. Brain 138, 2987–3002. 10.1093/brain/awv212 26220941 PMC4627353

[B100] Scarduzio M. Zimmermann C. N. Jaunarajs K. L. Wang Q. Standaert D. McMahon L. L. (2017). Strength of cholinergic tone dictates the polarity of dopamine D2 receptor modulation of striatal cholinergic interneuron excitability in DYT1 dystonia. Exp. Neurol. 295, 162–175. 10.1016/j.expneurol.2017.06.005 28587876 PMC5561742

[B36] Schmitz C. Hof P. R. (2005). Design-based stereology in neuroscience. Neuroscience 130, 813–831. 10.1016/j.neuroscience.2004.08.050 15652981

[B37] Schneider C. A. Rasband W. S. Eliceiri K. W. (2012). NIH image to ImageJ: 25 years of image analysis. Nat. Methods 9, 671–675. 10.1038/nmeth.2089 22930834 PMC5554542

[B38] Sciamanna G. Hollis R. Ball C. Martella G. Tassone A. Marshall A.. (2012a). Cholinergic dysregulation produced by selective inactivation of the dystonia-associated protein torsinA. Neurobiol. Dis. 47, 416–427. 10.1016/j.nbd.2012.04.015 22579992 PMC3392411

[B40] Sciamanna G. Tassone A. Mandolesi G. Puglisi F. Ponterio G. Martella G.. (2012b). Cholinergic dysfunction alters synaptic integration between thalamostriatal and corticostriatal inputs in DYT1 dystonia. J. Neurosci. 32, 11991–12004. 10.1523/jneurosci.0041-12.2012 22933784 PMC3471539

[B41] Sholl D. A. (1953). Dendritic organization in the neurons of the visual and motor cortices of the cat. J. Anat. 87, 387–406. 13117757 PMC1244622

[B42] Song C. H. Bernhard D. Bolarinwa C. Hess E. J. Smith Y. Jinnah H. A. (2013). Subtle microstructural changes of the striatum in a DYT1 knock-in mouse model of dystonia. Neurobiol. Dis. 54, 362–371. 10.1016/j.nbd.2013.01.008 23336980 PMC3628999

[B43] Standaert D. G. (2011). Update on the pathology of dystonia. Neurobiol. Dis. 42, 148–151. 10.1016/j.nbd.2011.01.012 21220015 PMC3073692

[B44] Tadic V. Kasten M. Brüggemann N. Stiller S. Hagenah J. Klein C. (2012). Dopa-responsive dystonia revisited: diagnostic delay, residual signs, and nonmotor signs. Arch. Neurol. 69, 1558–1562. 10.1001/archneurol.2012.574 22986512

[B45] Tepper J. M. Bolam J. P. (2004). Functional diversity and specificity of neostriatal interneurons. Curr. Opin. Neurobiol. 14, 685–692. 10.1016/j.conb.2004.10.003 15582369

[B46] Tepper J. M. Tecuapetla F. Koos T. Ibanez-Sandoval O. (2010). Heterogeneity and diversity of striatal GABAergic interneurons. Front. Neuroanat. 4:150. 10.3389/fnana.2010.00150 21228905 PMC3016690

[B47] Tepper J. M. Wilson C. J. Koós T. (2008). Feedforward and feedback inhibition in neostriatal GABAergic spiny neurons. Brain Res. Rev. 58, 272–281. 10.1016/j.brainresrev.2007.10.008 18054796 PMC2562631

[B48] Vitek J. L. (2002). Pathophysiology of dystonia: a neuronal model. Mov. Disord. 17, S49–S62. 10.1002/mds.10142 11948755

[B49] West M. J. (1999). Stereological methods for estimating the total number of neurons and synapses: issues of precision and bias. Trends Neurosci. 22, 51–61. 10.1016/s0166-2236(98)01362-9 10092043

[B50] West M. J. Slomianka L. Gundersen H. J. (1991). Unbiased stereological estimation of the total number of neurons in thesubdivisions of the rat hippocampus using the optical fractionator. Anat. Rec. 231, 482–497. 10.1002/ar.1092310411 1793176

[B51] Wijemanne S. Jankovic J. (2015). Dopa-responsive dystonia—clinical and genetic heterogeneity. Nat. Rev. Neurol. 11, 414–424. 10.1038/nrneurol.2015.86 26100751

[B52] Wilson B. K. Hess E. J. (2013). Animal models for dystonia. Mov. Disord. 28, 982–989. 10.1002/mds.25526 23893454 PMC3728703

[B53] Zhang L. Yokoi F. Parsons D. S. Standaert D. G. Li Y. (2012). Alteration of striatal dopaminergic neurotransmission in a mouse model of DYT11 myoclonus-dystonia. PLoS One 7:e33669. 10.1371/journal.pone.0033669 22438980 PMC3306281

